# Surgical management of carotid stump syndrome

**DOI:** 10.1016/j.jvscit.2023.101342

**Published:** 2023-10-03

**Authors:** Leah Lucero, Darian Siddhartha Dhawan, Leigh Ann O'Banion

**Affiliations:** Department of Surgery, University of California, San Francisco, Fresno, CA

First reported in the literature in the 1970s, the phenomenon termed “carotid stump syndrome” is defined by persistent cerebral ischemic events in the setting of an ipsilateral internal carotid artery (ICA) occlusion. The rare syndrome is hypothesized to occur due to embolism from the residual ICA stump into the middle cerebral artery territory via the external carotid artery. Treatment, although limited by case report experiences, has traditionally been ligation of the ICA with endarterectomy and patching of the common and external carotid arteries. The present patient provided written informed consent for the report of her clinical data and surgical video (Supplementary Video, online only).

## Patient background

Our patient is a 76-year-old woman who suffered a left-sided middle cerebral artery stroke with resulting right hemiparesis in 2019. Imaging after her initial stroke showed complete occlusion of the left ICA. She was medically managed with aspirin and clopidogrel. Subsequently, she presented on three separate occasions with recurrent hemiparesis and evidence of ongoing embolic strokes shown on magnetic resonance imaging. The magnetic resonance imaging scans taken during the patient's initial admission showed complete occlusion of the left ICA. Magnetic resonance angiography was also performed, which showed findings classic for carotid stump syndrome, including multiple large collateral vessels from the external carotid artery filling the ipsilateral hemisphere and reconstitution of the distal most segment of the supraclinoid left ICA, which was filling via contralateral perfusion. In addition, turbulent flow was visualized in the stump of the ICA on digital subtraction angiography. A cerebral angiogram (which was performed on a subsequent admission) showed occlusion of the left ICA at its origin, with a wisp of flow and no distal reconstitution. Attempts made to cross the lesion by our neurointerventional colleagues were unsuccessful. An attempt at crossing such occlusion poses an increased risk of thromboembolism without any added benefit; this was performed before our patient was referred to vascular surgery. Thus, we cannot comment on the decision or thought process. Due to ongoing symptoms and evidence of progressive embolic infarcts of the left hemisphere, in addition to the classic imaging findings, we diagnosed carotid stump syndrome. She was brought to the operating room for planned transection and ligation of her ICA with external carotid endarterectomy and patch angioplasty.

## Description of procedure

After making an incision along the anterior border of the sternocleidomastoid muscle, dissection was carried down to the carotid sheath. After opening the sheath, the jugular vein was exposed and the facial vein suture ligated and divided. The carotid artery was identified and the common and distal internal and external carotid arteries were encircled with vessel loops. The superior thyroid artery was identified and encircled with a free 2-0 silk tie. The patient was then systemically heparinized. Three minutes after heparin administration, the external and superior thyroid arteries were controlled. The common carotid artery and ICA were subsequently controlled. No decrease in cerebral oximetry occurred with clamping, and we elected not to use a shunt during the case.

The ICA was sharply transected using a no. 11 blade ∼1 cm distal to its origin. No back bleeding occurred from the ICA, which confirmed its occlusion. No atherosclerotic or thrombotic debris was visible within the ICA. We then oversewed the stump of the ICA with 5-0 Prolene suture and applied two large clips. The oversewing was done very carefully to balance the risk of stenosing the flow channel vs the risk of creating a new potential area for embolization. We then extended the arteriotomy with Potts scissors onto the external carotid artery and then down onto the common carotid artery, trimming away any excess tissue. We inspected the orifice of the external and common carotid arteries and, again, found no visible atherosclerotic or thrombotic material. Minimal plaque was present, consistent with the previous imaging findings. Because the ICA was occluded, we considered closing the external and common carotid arteries primarily. However, we elected to perform bovine patch angioplasty in an attempt to avoid narrowing the external carotid artery, because our patient was female with small vessels, and we believed she had a higher risk of stenosis with primary closure. We used a 0.8 × 8-cm LeMaitre bovine pericardial patch, which was sewn in place using 6-0 Prolene suture. Before completing the anastomosis, we back bled both the external and superior thyroid arteries and confirmed good inflow from the common carotid artery. We then flushed with heparinized saline, completed the anastomosis, and restored flow to the external carotid artery. Next, we obtained hemostasis and administered 30 mg of protamine to reverse the residual effects of the heparin. Once we were satisfied with the hemostasis, a Jackson-Pratt drain was placed in the surgical bed, and the incision was closed with 3-0 Vicryl suture to the platysma and a 4-0 Monocryl subcuticular suture to the skin. Finally, 2-octyl cyanoacrylate glue (Dermabond; Ethicon) was applied to the skin. The patient was neurologically intact on awakening and in recovery. She was discharged home on postoperative day 1.

## Patient follow-up

The patient had an uneventful postoperative course and recovery. She remains asymptomatic with a patent common carotid artery and external carotid artery with no evidence of hemodynamically significant stenosis. More than 2 years have passed since the operation was performed in February 2021.

## Discussion

Although a recognized phenomenon, the optimal management of carotid stump syndrome has been a source of debate.^1^ Comparative studies have evaluated the risks and benefits of medical vs surgical treatment (with the surgical standard of care ICA exclusion and external carotid artery endarterectomy).^2^ However, due to the rare prevalence of this syndrome, there is a paucity of well-powered studies to support either approach. The case highlighted in the Supplementary Video (online only) adds to the growing body of research supporting surgical intervention as a safe, effective treatment of carotid stump syndrome—especially for patients with recurrent ischemic events despite optimal medical management.

## Disclosures

None.

## Appendix (online only)

Supplementary Video (online only) Surgical management of carotid stump syndrome. *MCA,* Middle cerebral artery; *MR,* magnetic resonance.
